# Supplementary material for: Dissociable effects of emotional stimuli on electrophysiological indices of time and decision-making
Source: PLoS One. 2022 Nov 17;17(11):e0276200. doi: 10.1371/journal.pone.0276200 (PMC9671475; doi:10.1371/journal.pone.0276200)
Supplement: Attachment — Submitted filename: Response to Reviewers.docx [file pone.0276200.s001.docx]

We thank the reviews for their helpful comments in the improvement of our manuscript. We have addressed each comment individually and believe that it has enhanced the organization and understanding of our paper. Please see below for individual responses in blue and see the manuscript for changes and corrections which are indicated by red text.

Journal Requirements:

When submitting your revision, we need you to address these additional requirements.

1. Please ensure that your manuscript meets PLOS ONE's style requirements, including those for file naming. The PLOS ONE style templates can be found at

https://journals.plos.org/plosone/s/file?id=wjVg/PLOSOne_formatting_sample_main_body.pdf and

https://journals.plos.org/plosone/s/file?id=ba62/PLOSOne_formatting_sample_title_authors_affiliations.pdf.

We have addressed the formatting requirements as displayed in the PLOS ONE style templates.

2. We note that you have stated that you will provide repository information for your data at acceptance. Should your manuscript be accepted for publication, we will hold it until you provide the relevant accession numbers or DOIs necessary to access your data. If you wish to make changes to your Data Availability statement, please describe these changes in your cover letter and we will update your Data Availability statement to reflect the information you provide.

We will provide the necessary information for the data repository upon acceptance and understand that it will be held for publication until it has been provided.

3. Please include your full ethics statement in the ‘Methods’ section of your manuscript file. In your statement, please include the full name of the IRB or ethics committee who approved or waived your study, as well as whether or not you obtained informed written or verbal consent. If consent was waived for your study, please include this information in your statement as well.

We have verified that the methods section includes the full ethics statement as well as the full name of the IRB committee that approved our study.

4. We note that Figure 2 includes an image of a participant in the study.

As per the PLOS ONE policy (http://journals.plos.org/plosone/s/submission-guidelines#loc-human-subjects-research) on papers that include identifying, or potentially identifying, information, the individual(s) or parent(s)/guardian(s) must be informed of the terms of the PLOS open-access (CC-BY) license and provide specific permission for publication of these details under the terms of this license. Please download the Consent Form for Publication in a PLOS Journal (http://journals.plos.org/plosone/s/file?id=8ce6/plos-consent-form-english.pdf). The signed consent form should not be submitted with the manuscript, but should be securely filed in the individual's case notes. Please amend the methods section and ethics statement of the manuscript to explicitly state that the patient/participant has provided consent for publication: “The individual in this manuscript has given written informed consent (as outlined in PLOS consent form) to publish these case details”.

If you are unable to obtain consent from the subject of the photograph, you will need to remove the figure and any other textual identifying information or case descriptions for this individual.

We appreciate the concern regarding figure 2; however, we want to clarify that the image being referred to in figure 2 is an example of the face stimuli that was used in the paradigm. The source of these images have been cited in the manuscript.

Reviewers' comments:

Reviewer's Responses to Questions

**Comments to the Author**

1. Is the manuscript technically sound, and do the data support the conclusions?

The manuscript must describe a technically sound piece of scientific research with data that supports the conclusions. Experiments must have been conducted rigorously, with appropriate controls, replication, and sample sizes. The conclusions must be drawn appropriately based on the data presented.

Reviewer #1: Partly

Reviewer #2: Yes

2. Has the statistical analysis been performed appropriately and rigorously?

Reviewer #1: Yes

Reviewer #2: Yes

3. Have the authors made all data underlying the findings in their manuscript fully available?

The [PLOS Data policy](http://www.plosone.org/static/policies.action#sharing) requires authors to make all data underlying the findings described in their manuscript fully available without restriction, with rare exception (please refer to the Data Availability Statement in the manuscript PDF file). The data should be provided as part of the manuscript or its supporting information, or deposited to a public repository. For example, in addition to summary statistics, the data points behind means, medians and variance measures should be available. If there are restrictions on publicly sharing data—e.g. participant privacy or use of data from a third party—those must be specified.

Reviewer #1: Yes

Reviewer #2: Yes

4. Is the manuscript presented in an intelligible fashion and written in standard English?

PLOS ONE does not copyedit accepted manuscripts, so the language in submitted articles must be clear, correct, and unambiguous. Any typographical or grammatical errors should be corrected at revision, so please note any specific errors here.

Reviewer #1: No

Reviewer #2: Yes

5. Review Comments to the Author

Please use the space provided to explain your answers to the questions above. You may also include additional comments for the author, including concerns about dual publication, research ethics, or publication ethics.

Reviewer #1: Thank you for giving me the opportunity to review this paper.

The paper reports a behavioural and an ERP experiment. The behavioural experiment established the influences of emotional faces on the chosen visual temporal task with a relatively larger sample (n=61). The EEG experiment studied the ERPs at different time points with a smaller sample (n=31). The setup is understandable considering the availability of equipment and the difficulty in the experimental setup.

The experiments employed a specific procedure presenting two emotional faces in a trial, before and after the visual temporal stimuli. The emotional faces before the stimuli were expected to influence attention and arousal to the perceptual period, while the faces after the stimuli provided information on how they affect the decision process. It is a novel setup to me.

The authors shall address the following concerns before the manuscript shall be considered for publication.

1. The authors shall provide a more detailed account of the data exclusion criteria. On page 11, “three participants were removed because their behavioural data was more than two standard deviations from the mean”, but it does not mention which parameters. A similar comment applied to the participant removed due to ERP characteristics that it is unclear which components or just the grand average. Further, the two participants excluded in the RT analyses (page 14) also required further elaboration.

We thank the reviewer for bringing our attention to the lack of detail in regard to participant exclusions. We have addressed the issues in the manuscript. For the behavioral data we added additional information regarding which parameter led to the exclusion. We additionally added more detail about the ERP data exclusions. As for the RT analysis we included the following information “...due to missing data caused by an issue with the data files.” to further elaborate the reason for exclusion from the analysis.

2. On page 12, the authors reported using ICA to detect and remove artefacts relating to eye blinks and noise. A more detailed description to the cleaning pipeline will be appreciated, and please cite the references if the authors followed any published protocols.

Thank you for this suggestion, we have included in the text that artifacts were visually inspected and removed as well as included the citation Jung et al., 1997..

3. Please provide detailed descriptions of the instructions to the participants. For example, it is unclear how the participants understand the ‘long’ and ‘short’ responses. Is there any practice block?

We have addressed this issue by adding in the additional detail regarding instructions participants received and whether or not there was a practice block. We added the line “ Participants did not complete any practice trials and were instructed to judge the duration of the temporal stimulus on each trial as short or long based on all other durations experienced.” in the methods section of the behavioral study.

4. Please provide the measurement units to the tables. Should it be milliseconds (ms) for BP and RT?

We appreciate the reviewer pointing out this oversight. We have addressed the issue and the tables now reflect units of measurement.

5. Please provide effect sizes to the behavioural effects of the EEG study though they are not significant.

This has been addressed and effect sizes have been included in the manuscript.

6. Kindly elaborate on how the amplitude analyses were done. For example, do the authors use local peak or mean amplitude in the EPOCH?

We added the statement “All epochs were analyzed using the mean amplitude.” to the methods section of the manuscript.

7. It would be nice to have a EEG map to show the chosen electrodes for the ERP analyses of various components. Further, there appears to be an overlap of different ERPs in time. It would be nice if there is a timeline in a similar format to Figure 2 but shows the duration of different EPOCHs. It is also unclear how the potential overlap may influence the ERP analyses. The authors shall critically discuss this possibility.

Thank you for your input, we have added an electrode map of chosen electrodes to the topography maps in figures 4, 5, and 6. As for the epoch overlaps, the only overlap would be for the CNV ERP for the two shortest durations; however, the time frame of each epoch we analyzed does not overlap with any other epoch time frame. We therefore believe that there is no issue to address in regards to the possibility of overlap in the ERP analysis.

8. The authors describe arousal and attention influence the pacemaker and the gate, respectively, in affecting the visual temporal task. A more critical analysis will be appreciated. Further, on page 19, the authors describe the “gate opening wider via increased attention”. Instead of increased attention, it appears to be more common to describe attention as being diverted. Also, the gate is described as flickered or delayed in its opening instead of opening wider.

We thank the reviewer for bringing our attention to this issue. We have added in more text in regard to the pacemaker or gate influences. We also corrected the issue in the discussion about the gate opening wider to be more clear.

9. The argument in the discussion about LPCt appears to be weak. It contrasts the LPCt amplitudes between the Neut>Neg and Neut>Neut condition and claims a marginally significant difference in the amplitudes among the ‘short’ responses. However, there is no behavioural difference between these conditions in Experiments 1 and 2. There is, perhaps, insufficient support for the effect of negative faces on the decision process stage.

We agree with the review that the LPCt discussion is weak; however we do believe it is worth mentioning the effect we observed and potential reasons for that difference. In order to address this issue we added the following text to the end of the LPCt discussion. “Although the behavioral data does not fully support these conclusions, we believe the EEG results do suggest that the LPCt differences do have something to do with decision-making; however, it is unclear exactly what from these data alone.”

10. Partially follow up on #3, the authors shall probably acknowledge the limitation of using a fixed ISI (500ms) similar to their physical bisection point. Depending on the experimental protocols, the participants may use the ISI to help the judgement.

We have addressed this issue in the methods section of the text with the following statement. “We used a fixed inter-stimulus interval (ISI) which could lead to subjects using it as a standard in which to compare the temporal interval to; however, if this were the case we would expect the BP to be closer to the ISI (500ms) which is not the case nor would it change the findings of the study.”

Reviewer #2: The aim of the present study is to determine whether the effect of emotion on time perception results from an alteration of the clock speed or from a decision bias. To distinguish between the two hypotheses, neutral or negative (angry) faces were presented before and/or after the visual stimulus whose duration was evaluated by participants. Two experiments were conducted. A first behavioural experiment revealed that the stimulus duration was overestimated when preceded by an angry face in line with an increase of the clock speed. A second EEG experiment revealed that the contingent negative variation (CNV) measured during the visual stimulus was enhanced when preceded by an angry face still in line with an increase of the clock speed. Furthermore, the late positive component of timing (LPCt) following 'short' responses was reduced when an angry face was presented after the visual stimulus suggesting that decision processes were also altered by emotion.

This is a well-designed study based on the combination of two approaches (behavioural and EEG) addressing a straightforward issue. Although results are not as clear as hypotheses, I would suggest that the paper would be publishable as it proposes an original paradigm allowing to carefully examine the effect of emotion on time processing which would aid in a better understanding of the underlying neural and cognitive mechanisms. However, I have several concerns with the results and the way they are discussed as well as with some aspects of the methodology. Here are my comments and suggestions:

Major Points:

1. As noted by the authors, in the EEG experiment, ERPs measured during the visual stimulus were expected to differ between the neg-neut and the neut-neut conditions but not between the neut-neg and the neut-neut conditions, since at this moment of the trial, the two conditions are identical. However, the CNV amplitude was significantly larger and the N1 amplitude tended to be larger for neut-neg trials than for neut-neut trials. The authors proposed that the larger CNV amplitude for the neut-neg condition would only concern trials following a neg-neut trial because after a neg-neut trial, participants would predict a negative face in first position and thus, a neutral face presented in first position would produce a positive prediction error which would induce an increased CNV amplitude. However, the same logic seems to apply for both neut-neg and neut-neut trials following a neg-neut trial (note also that, according to this explanation in terms of predictive error, a negative face presented in first position should produce a negative prediction error and thus and a duration underestimation and a lower CNV amplitude). Alternatively, the authors proposed that, after a neg-neut trial, when a neutral face is presented in first position, participants would anticipate a negative face in second position and that such anticipation would induce an increased CNV amplitude. However, here again, the same reasoning seems to apply equally for both neut-neg and neut-neut trials. These different explanations thus appear as confusing. As there is no expected difference between neut-neg and neut-neut conditions for the N1 and the CNV measured during the visual stimulus, could these two conditions be grouped together and the statistical analyses be performed between trials starting with a negative face vs trials starting with a neutral face?

We agree with the reviewer and have corrected the manuscript to reflect the suggestion. We opted to instead compare between trials starting with a negative face first with those in which a neutral face came first. Results of the analysis have been updated in the manuscript; however, we are leaving the tables and figures as initially analyzed for completeness.

2. The paradigm is not symmetric due to the absence of neg-neg condition. Thus, when the first face is negative, participants can predict than the second face will be neutral as there is not neg-neg trial. By contrast, when the first face is neutral, participants cannot anticipate whether the second face will be neutral of negative. Participants would thus be in a "predictive context" following a negative face and a in an "unpredictive context" following a neutral face. How exclude the fact that the longer duration estimates and the larger CNV amplitude observed for a visual stimulus preceded by an angry face would be due to the context (predictive vs unpredictive) rather than to the emotion per se (angry vs neutral)?

We agree that the addition of a neg-neg condition could have been beneficial to the study; however, we elected not to include it as we were only looking at the effects of a singular negative face before and after a stimulus rather than all possible combinations. We would like to note that if participants were in a predictive context following a negative face we would expect the CNV to be lower rather than higher since there is no anticipation of an upcoming negative face, which is not the case. Therefore, we believe the paradigm to be sufficient for the research question in this case.

Minor Points:

1. Concerning the first behavioural experiment, how explain the important loss of participants from the inclusion (61) to the statistical analyses (39 participants)? It is not clear whether this behavioural experiment was an online experiment and whether participants performed the task at home in an uncontrolled context. A bisection task was used but there is no information about the anchors (300 and 900ms?) and the number of presentations of each of them.

We thank the reviewer for bringing our attention to this issue. Within the text we explain that 14 participants did not complete the study, 3 were excluded due to always responding short despite no matter the duration, and 5 were excluded due to BP’s being more than two standard deviations from the mean. We have clarified in the text that this was an online study without compensation and we believe this led to the large number not completing the study as well as the poor performance. As for the anchors, we provided no explicit anchors and instead used the “partition” method employed by our lab and others (cf. Wearden & Ferrera, 1995) in which subjects simply judge each interval relative to all previously experienced intervals; prior work has demonstrated identical performance on this version of the task to those with explicit anchors.

Wearden, J. H., & Ferrara, A. (1995). Stimulus spacing effects in temporal bisection by humans. The Quarterly Journal of Experimental Psychology, 48(4), 289-310.

2. Faces were presented during 600ms which corresponds approximately to the bisection point. Why did you use this duration? Is it possible that participants actually compared the duration of the visual stimulus with that of the faces rather than with that of the memorized anchors?

We thank the reviewer for questioning the duration of faces; however, we chose the 600ms duration because it corresponded to the bisection point. We believe that, since a duration had to be chosen, this would cause the least amount of confusion to the participants. In the verbal instructions participants were informed that the faces always appeared for the same amount of time (but not the temporal stimulus) so they need not pay attention to their duration but rather only focus on the temporal stimulus and whether or not it was short or long based on all other durations experienced. However, if subjects were using the face as a reference we would expect that in the neg-neut condition participants would be more likely to say short which would lead to a lower CNV because the face itself would appear longer making the temporal interval appear relatively shorter. We therefore believe that the interval for which the face presented would change the results of the study..

3. Although no significant effect of condition on the bisection point (BP) was observed in the EEG experiment, did you examine and observe correlations between behavioural and ERP indices? For example, is the difference of BP between neg-neut and neut-neut conditions correlated with the difference of CNV amplitude between the two same conditions? Did you also examine and observe correlations between the different ERP indices? For example, is the difference of N170 amplitude between negative and neutral faces presented in first position correlated with the difference of CNV amplitudes between trials starting with a negative face and trials starting with a neutral face?

We appreciate the reviewer’s suggestion to consider additional analyses such as correlations between the behavioral data and ERP indices. We did look into such correlations but unfortunately did not observe any significant correlations between behavioral data and ERP indices nor were there correlations between the ERP indices.

4. There is a problem with the sentence in lines 307 to 310. Concerning the results on the N170, how explain that amplitudes are larger for faces presented in second position than for faces presented in first position? Could this be due to neural suppression by repetition of the same face?

We have made the necessary corrections to the referred sentences and suggest that this finding could be due to neural suppression.

5. The sentence in lines 107 to 109 (marking the beginning of a new section concerning the investigation of time perception with EEG) could be placed at the beginning of the following paragraph.

We agree with the reviewer that this sentence would serve better as the beginning of the next paragraph and have made this change in the manuscript.

6. PLOS authors have the option to publish the peer review history of their article ([what does this mean?](https://journals.plos.org/plosone/s/editorial-and-peer-review-process#loc-peer-review-history)). If published, this will include your full peer review and any attached files.

If you choose “no”, your identity will remain anonymous but your review may still be made public.

**Do you want your identity to be public for this peer review?** For information about this choice, including consent withdrawal, please see our [Privacy Policy](https://www.plos.org/privacy-policy).

Reviewer #1: **Yes:** Li Wang On

Reviewer #2: No

While revising your submission, please upload your figure files to the Preflight Analysis and Conversion Engine (PACE) digital diagnostic tool, <https://pacev2.apexcovantage.com/>. PACE helps ensure that figures meet PLOS requirements. To use PACE, you must first register as a user. Registration is free. Then, login and navigate to the UPLOAD tab, where you will find detailed instructions on how to use the tool. If you encounter any issues or have any questions when using PACE, please email PLOS at figures@plos.org. Please note that Supporting Information files do not need this step.
