# Supplementary material for: Dissociable effects of emotional stimuli on electrophysiological indices of time and decision-making
Source: PLoS One. 2022 Nov 17;17(11):e0276200. doi: 10.1371/journal.pone.0276200 (PMC9671475; doi:10.1371/journal.pone.0276200)
Supplement: Attachment — Submitted filename: Response to Reviewers.docx [file pone.0276200.s002.docx]

PONE-D-22-09658R1

Dissociable effects of emotional stimuli on electrophysiological indices of time and decision-making

PLOS ONE

We thank the reviewers for their comments. We have addressed each comment individually in blue text. Please see the manuscript for changes indicated by red text.

Journal Requirements:

Please review your reference list to ensure that it is complete and correct. If you have cited papers that have been retracted, please include the rationale for doing so in the manuscript text, or remove these references and replace them with relevant current references. Any changes to the reference list should be mentioned in the rebuttal letter that accompanies your revised manuscript. If you need to cite a retracted article, indicate the article’s retracted status in the References list and also include a citation and full reference for the retraction notice.

We have reviewed are reference list and believe it to be complete and correct.

Reviewers' comments:

Reviewer's Responses to Questions

**Comments to the Author**

1. If the authors have adequately addressed your comments raised in a previous round of review and you feel that this manuscript is now acceptable for publication, you may indicate that here to bypass the “Comments to the Author” section, enter your conflict of interest statement in the “Confidential to Editor” section, and submit your "Accept" recommendation.

Reviewer #1: All comments have been addressed

2. Is the manuscript technically sound, and do the data support the conclusions?

The manuscript must describe a technically sound piece of scientific research with data that supports the conclusions. Experiments must have been conducted rigorously, with appropriate controls, replication, and sample sizes. The conclusions must be drawn appropriately based on the data presented.

Reviewer #1: Yes

3. Has the statistical analysis been performed appropriately and rigorously?

Reviewer #1: Yes

4. Have the authors made all data underlying the findings in their manuscript fully available?

The [PLOS Data policy](http://www.plosone.org/static/policies.action#sharing) requires authors to make all data underlying the findings described in their manuscript fully available without restriction, with rare exception (please refer to the Data Availability Statement in the manuscript PDF file). The data should be provided as part of the manuscript or its supporting information, or deposited to a public repository. For example, in addition to summary statistics, the data points behind means, medians and variance measures should be available. If there are restrictions on publicly sharing data—e.g. participant privacy or use of data from a third party—those must be specified.

Reviewer #1: Yes

5. Is the manuscript presented in an intelligible fashion and written in standard English?

PLOS ONE does not copyedit accepted manuscripts, so the language in submitted articles must be clear, correct, and unambiguous. Any typographical or grammatical errors should be corrected at revision, so please note any specific errors here.

Reviewer #1: Yes

6. Review Comments to the Author

Please use the space provided to explain your answers to the questions above. You may also include additional comments for the author, including concerns about dual publication, research ethics, or publication ethics. (Please upload your review as an attachment if it exceeds 20,000 characters)

Reviewer #1: The authors have addressed most comments. Both reviewers asked questions about presenting fixed durations before the target (i.e. a 600ms face and a 500ms blank screen). Another common concern was the lack of practice and explicit anchors. The authors’ responses are satisfactory. They have included some elaborations in the revised manuscript while leaving out some in the reply letter only. A brief paragraph discussing these methodological issues will be appreciated.

We thank the reviewer for drawing our attention to the fact that we did not include these discussions in the manuscript itself. We have corrected this issue by adding the following to the methods section: “Additionally, we chose the 600ms duration since it corresponded to the bisection point of the intervals used and believed that this would cause the least amount of confusion to participants. We did inform participants that the faces always appeared for the same amount of time so they need not pay attention to the duration of those faces but only focus on the temporal stimulus. If subjects were using the face as a reference, participants would be more likely to say short in the NegàNeut condition leading to a lower CNV which is not the case. Therefore, we believe that the duration for which the face was presented would not change the results of the study.”

There are two minor issues in the figures.

1. The legends in the new topography maps overlap and cannot be read clearly (Figure 4 and 5).

We thank the reviewer for catching this formatting issue. We have fixed the figures and uploaded corrected ones.

2. Figure 5 caption describes *1 and *2, but the figure shows *2 and *3.

We appreciate the reviewers attention to detail and catching this error. We have updated the figure caption with the correct information.

7. PLOS authors have the option to publish the peer review history of their article ([what does this mean?](https://journals.plos.org/plosone/s/editorial-and-peer-review-process#loc-peer-review-history)). If published, this will include your full peer review and any attached files.

If you choose “no”, your identity will remain anonymous but your review may still be made public.

**Do you want your identity to be public for this peer review?** For information about this choice, including consent withdrawal, please see our [Privacy Policy](https://www.plos.org/privacy-policy).

Reviewer #1: **Yes:** Wang On LI

[NOTE: If reviewer comments were submitted as an attachment file, they will be attached to this email and accessible via the submission site. Please log into your account, locate the manuscript record, and check for the action link "View Attachments". If this link does not appear, there are no attachment files.]

While revising your submission, please upload your figure files to the Preflight Analysis and Conversion Engine (PACE) digital diagnostic tool, <https://pacev2.apexcovantage.com/>. PACE helps ensure that figures meet PLOS requirements. To use PACE, you must first register as a user. Registration is free. Then, login and navigate to the UPLOAD tab, where you will find detailed instructions on how to use the tool. If you encounter any issues or have any questions when using PACE, please email PLOS at figures@plos.org. Please note that Supporting Information files do not need this step.
